# Burnout Syndrome in professionals working in mental health

**DOI:** 10.1192/j.eurpsy.2023.940

**Published:** 2023-07-19

**Authors:** M. Valverde Barea, C. Mata Castro, P. Vargas Melero, M. O. Solís Correa, F. Cartas Moreno

**Affiliations:** Hospital Universitario Jaén, Jaén, Spain

## Abstract

**Introduction:**

Burnout syndrome or professional exhaustion is defined as feeling burned out, exhausted, overloaded, exhausted. It is a syndrome characterized by emotional exhaustion, depersonalization and low personal fulfillment. This clinical syndrome was first described in 1974 by Herbert Freudenberger, a psychiatrist, who defined burnout as “the depletion of energy experienced by professionals when they feel overwhelmed by the problems of others.” Mental Health is one of the specialties with the greatest emotional exposure due to all the circumstances that surround these professions, to maintain health in its 3 axes: physical, mental and social well-being as defined by the WHO

**Objectives:**

The objective of the study is to determine the presence of Burnout Syndrome in Mental Health professionals through the Maslach Burnout Inventory (MBI) questionnaire.

**Methods:**

An observational, descriptive and cross-sectional study is carried out. The people included in the study were the health personnel of the Mental Health Clinical Management Unit (psychiatrists, administrative personnel, nursing assistants, nursing personnel, social workers and psychologists, and training personnel) who wanted to participate in the study. Carrying out the MBI questionnaire and sociodemographic data.

**Results:**

In our study we have a sample of 59 people. Regarding the sociodemographic data, we have 45 women and 14 men. Regarding the results after correcting the MBI questionnaire, we found that 4 professionals presented Burnout Syndrome (a psychiatrist and a 4th year psychiatry resident intern of psychiatry), 35 professionals presented tendency to suffer from Burnout since one of the three areas measured by the questionnaire was affected and 15 did not suffer from Burnout. Regarding the domains, we obtain that emotional exhaustion is the area, together with low personal achievement, that is most affected in the professionals of the community mental health unit, 23 and 22 professionals, respectively. Depersonalization is present at 12. Professionals with temporary contracts presented greater emotional exhaustion and low personal accomplishment. Professionals with permanent contracts show greater emotional exhaustion. Among the professionals in training, low personal achievement and depersonalization stand out. The 4 professionals who present burnout syndrome are married women and 3 of them with temporary contracts.

**Conclusions:**

The results obtained show the presence of Burnout Syndrome and a high tendency to develop it among the professionals of the Mental Health Unit. In relation to the data, we must reassess the care systems for professionals and prevent the causes that can lead professionals who are starting their professional career to develop burnout in normal situations that can lead to collapse in extraordinary circumstances such as the COVID-19 pandemic.

**Disclosure of Interest:**

None Declared

